# Patient-informed exploration of the aftermath of a diagnostic problem or mistake based on results of a national survey

**DOI:** 10.3389/frhs.2024.1474073

**Published:** 2024-11-28

**Authors:** Kelly T. Gleason, Christina T. Yuan, Helen Haskell, Michelle A. Anderson, Jane A. Evered, Kathryn M. McDonald

**Affiliations:** ^1^ School of Nursing, Johns Hopkins University, Baltimore, MD, United States; ^2^ Health Policy and Management, Bloomberg School of Public Health, Johns Hopkins University, Baltimore, MD, United States; ^3^ Mothers Against Medical Error, Columbia, SC, United States; ^4^ Community Advisory Panel, New England Precision Medicine Consortium, All of Us Research Program, Boston, MA, United States; ^5^ Department of Family Medicine and Community Health, University of Wisconsin-Madison, Madison, WI, United States

**Keywords:** diagnostic safety, patient safety, patient engagement, family engagement, quality improvement

## Abstract

**Introduction:**

Despite the prevalence and devastating consequences of diagnostic breakdowns, there have been minimal efforts to systematically collect patient insight into diagnostic problems and mistakes. Collaborating with patient advocates to guide how patient-derived insights are interpreted and used is a critical, yet often overlooked, approach to identifying actionable solutions that speak to patients' priorities.

**Objective:**

We collaborated with patient advocate co-authors to guide our understanding of findings from a mixed methods survey on diagnostic problems and mistakes, and report implications for patient engagement at three levels of action: (1) individual level before, during, after encounters (*micro*); (2) within health service delivery systems (*meso*); and (3) policy advocacy (*macro*).

**Methods:**

Our research team applied narrative elicitation methods to conduct a novel survey about Americans' diagnostic experiences in a national, population-based survey. We shared early results with patient co-authors who highlighted the importance of further exploring how health systems and clinicians address the aftermath of diagnostic mishaps. Based on their input, we summarized the quantitative and qualitative survey results about the aftermath and worked with our patient co-authors to explore how findings might inform actionable next steps, including efforts to catalyze patient action, quality improvement efforts, and policy reform.

**Results:**

Of the 3,684 survey respondents, about a third (33.0%, 1,216/3,684) of screened households reported diagnostic problems and mistakes in the past four years involving either themselves (18.9%, 697/3,684) or someone close to them (14.1%, 519/3,684). In the aftermath of a diagnostic mishap, over a third reported that someone in the healthcare setting where the mistake occurred acknowledged the mistake (35.9%, 432/1,204). In qualitative findings, reports that the health system “did nothing” surfacing as the most common response. Patient co-authors confirmed the results resonated with their experiences and emphasized the need for health systems to take accountability when a mishap occurs and to take follow-up actions to prevent future mishaps.

**Discussion:**

Patients and care partners not only want and deserve acknowledgement of diagnostic problems or mistakes in their own care, they also want assurance that steps are being taken to prevent similar events from happening to others. Across micro-, meso-, and macro-levels of action, working with patients to understand and act on contributors to diagnostic breakdowns is aligned with high-reliability organizing principles.

## Introduction

The need to improve diagnosis is urgent: diagnostic mistakes are the most deadly and costly of medical errors (1). Patient and care partner, a family or friend who partners in a patient's care, (hereon referred to as “patients”) engagement is essential to healthcare transformation (2), including diagnostic quality improvement efforts (3). However, the invaluable insights patients can offer about breakdowns in the diagnostic process (4) and about other factors that adversely impact their diagnostic experiences (5–7) are not regularly collected or used for quality improvement and high reliability organizing (8–10). The latter paradigm, emerging from studies of high levels of safety in high hazard industries (including healthcare delivery), recognizes the importance of deference to expertise, defined as appreciating that the people closest to the work are often the most knowledgeable about that work (10). In healthcare delivery, there is no question that patients are among the people closest to the work.

Many in the healthcare community recognize the need to engage patients and care partners in the design and conduct of research and quality improvement efforts to drive improved care and high reliability performance in the healthcare system. While patient engagement in programs of research focused on treating or managing a specific health condition is increasingly common (11), patient engagement in diagnostic improvement efforts is less common and not yet applied as a critical and natural source of expertise for high reliability organizing for diagnostic safety. The nature of diagnosis adds to the challenge of patient engagement in improvement efforts: diagnosis often occurs over time across multiple settings with multiple clinicians, and reasons for diagnostic mistakes are often multifactorial. The complex nature of diagnosis makes identifying a main cause difficult even for expert clinicians and is aligned with high reliability organizing principles which eschew main cause thinking (e.g., an error occurred because of one clinician's mistake) in lieu of a systems approach (e.g., an error occurred because of a host of factors). However, these same characteristics of diagnosis and its variegated contexts make patient input even more essential: only patients know their full diagnostic trajectory, and this knowledge is critical for understanding where breakdowns may have occurred and informing individual, system-level, and policy-level efforts to improve quality of care.

The value of engaging patients in programs of research for diagnostic quality is clear. Best practice consensus is that patients should be engaged across the continuum of the research process, from project inception through dissemination (11). In practice, however, engagement activities have been typically concentrated in the bookends of the research endeavor, including the beginning (in which patients are engaged in the formative work that informs data collection efforts) and the end (in which patients are engaged in helping to translate and disseminate findings). A stage that often goes overlooked is engaging patients in the analysis of data, despite increasing recognition that patient partners lead to more comprehensive interpretation of findings, and more community-aligned solutions (2, 12). Recent patient-driven efforts, including the citizen scientist community for long COVID (12) and the Quantified Self Community (13), illustrate how meaningful patient engagement and leadership throughout the research process can lead to more comprehensive solutions. The principles of the citizen science community have yet to be robustly applied in improvement initiatives to address diagnostic errors, problems, and mistakes.

Drawing from our narrative elicitation project and recently fielded national survey on patient-reported diagnostic problems and mistakes, we report analytic findings for a key domain of patient experiences with diagnostic failures chosen by our patient advocate co-authors, and together derive learnings and potential actions from these findings. This paper aims to report implications for patient engagement driven by our patient-informed analysis summarized at three levels of potential action: (1) individual level before, during, after encounters (micro); (2) within health service delivery systems (meso); and (3) policy advocacy (macro).

## Materials and methods

### Research team reflexivity

The authors include two patient advocates (HH, MA), two clinician-researchers (KG, JE), and two organizational researchers (CY, KM). All authors report personally experiencing diagnostic problems and mistakes (also abbreviated as diagnostic mishaps and inclusive of diagnostic errors) as patients and/or care partners.

The authors adopt a patient-centered, whole-person approach to research and hold the beliefs that patients are experts in their health experiences, and that care partners often have a close-in view of their loved one's health experiences. The authors hold the contextual constructivist perspective that meaning is constructed through participants' understandings, the co-authors' interpretations (typically referred to as the researchers' interpretations), the sociocultural context and the interpretations of the scientific community (14). Thus, we recognize that our identities and experiences play a role in shaping our research and reporting.

### Background of diagnostic problems and mistakes survey

We conducted a population-based survey drawing on the NORC AmeriSpeak® Panel, nationally representative sample in 2023. The survey was designed in part through work with a technical expert panel (TEP). Patient and care partner representatives, including co-authors, contributed to the TEP. We first pilot tested the survey on a sample of 671 respondents, using the open-ended descriptions of diagnostic experiences to verify characterization of diagnostic problems and mistakes. The survey is included in the Supplementary Material. The NORC AmeriSpeak® panel is a probability-based panel that provides sample coverage of approximately 97% of the U.S. household population. The study was approved by the institutional ethics boards at collaborating universities. Eligible participants were patients or care partners ages 18 or older with proficiency in English. Demographic data was collected from each participant: age, gender, race/ethnicity, and education. Of 3,684 individuals screened, those who had experienced diagnostic problems answered questions about one or more diagnostic experiences over the last four years in three iterations of the survey and in interviews with a subset. Participants who reported experiencing multiple problems and/or mistakes were guided to select a single problem and/or mistake for the survey responses. The resulting survey response rate was 26.5%, with 95.4% completing the entire set of questions. Rich data from more than 1,200 cases of lived experiences with diagnostic problems are available from this data source.

### Collaboration with patient co-authors to identify domain for further analysis

After fielding the survey, the research team met with our patient co-authors and presented early results from five key domains of experience where the patient (care partner) is an essential source of information including: (1) how well providers communicated throughout the diagnostic process, (2) how any personal attributes of the patient, such as background, culture, identity or health needs, made diagnostic experiences better or worse, (3) whether a clinician or other person was a reliable source of guidance and support during the diagnostic process, (4) types and duration of harms associated with diagnostic problems that persist and affect patient or family well-being, and (5) what happens in terms of remediation, compensation, or other efforts from the health system/clinical team to address the diagnostic problem or mistake. The patient co-authors were most interested in deeper analysis of the fifth domain, focused on what we termed as the “diagnostic aftermath”, and particularly emphasized current gaps in how clinicians and the health system learn from and address problems and mistakes.

Following the guidance from our patient co-authors, we conducted further exploration of the quantitative and qualitative data related to how the health system and providers addressed the aftermath of diagnostic mishaps. We examined responses to quantitative questions using summary statistics on whether the diagnostic problem or mistake was acknowledged by the healthcare team, whether the healthcare team apologized if an apology was necessary, and, if the patient (care partner) did not report the diagnostic problem or mistake to the healthcare team, why they did not report it (Table 1). We also preliminarily examined the qualitative responses to three open-ended survey questions related to what patients (care partners) perceived could have been done differently. We categorized the responses using a qualitative descriptive approach (15, 16) paired with inductive content analysis (17).

**Table 1:** Quantitative and qualitative survey questions examined to explore post-event response and resolution.

Quantitative	Qualitative
1) Did anyone in the healthcare settings where the diagnostic mistake or problem occurred acknowledge to [INSERT PRONOUN] that something had gone wrong? a. Yes b. No 2) Did [INSERT PRONOUN] receive an apology about the diagnostic problem? a. Yes b. No, and an apology would have been appropriate c. No, and circumstances didn't warrant an apology	1) “After [you/they] realized that there was a problem with the diagnosis, what, if anything, did the doctors and other clinicians do or say that **made things better**? This could include things that improved [your/their] health, [your/their] medical care, or how you/they] felt about the diagnostic experiences?” 2) “After [you/they] realized that there was a problem with the diagnosis, what, if anything, did the doctors and other clinicians do that **made things worse**? This could include anything that negatively impacted [your/their] health, [your/their] medical care, or how [you/they] felt about the diagnostic experiences?” 3) “After [you/they] realized there was a diagnostic problem, what, if anything, do [you/they] **wish had been done** by doctors, clinicians, or others in the healthcare system to improve the situation?”

The patient co-author insights were chiefly gathered based on 3 h of live interaction (a one-hour meeting with both co-authors together and then one hour-long meeting with both patient co-authors separately). The co-authors were sent the findings in advance to review, and the sessions were focused on ascertaining their interpretation of the findings, and their initial ideas around patient-level, healthcare system-level, and policy level solutions. Asynchronous communication occurred throughout for revisions to and confirmation of the insights included.

## Results

Of the 3,684 survey respondents, about a third (33.0%, 1,216/3,684) of screened households reported a diagnostic mishap in the past four years involving either themselves (18.9%, 697/3,684) or someone close to them (14.1%, 519/3,684). Of the 3,684 survey respondents, half were female (50.6%, 1,865/3,684) and the majority (62.6%, 2,307/3,684) identified as white, 12.8% identified as Black, and 17.9% as Hispanic. The majority of respondents reported that the healthcare delivery site where the mishap occurred did not acknowledge the mishap or offer an apology. For the engagement of patient co-authors in interpreting these emerging findings, we selected two preliminary categories from the qualitative analysis to focus our discussions, including: (1) post-event response experiences, and (2) post-event resolution experiences.

### Prevalence estimates of patient and clinician actions in the diagnostic aftermath shared with patient co-authors

Over a third of survey respondents reported that someone in the healthcare setting where the mistake occurred acknowledged the mistake (35.9%, 432/1,204). Excluding those who reported that an apology was not necessary (*n* = 377), about one-third of respondents reported receiving an apology (33.0%, 275/839). Those who did not report the diagnostic problem (*n* = 530) identified the following reasons for not reporting: (1) they did not think it would do any good (43.8%, 232/530), (2) there was no way to do so anonymously (21.5%, 114/530), and (3) because they did not want to get anyone in trouble (17.4%, 92/530). Only one-tenth reported that someone on the care team offered information about a formal review or investigation to determine what caused the problem (10.9%, 135/1,216); more reported that they received an explanation of actions they were taking to prevent similar diagnostic problems (18.8%, 220/1,216). About a third reported that someone on the care team spoke openly and truthfully about the problems (426/1,216, 34.5%).

In the open-ended comments, “did nothing” was the most commonly noted post-event action by providers in the qualitative responses and was identified 325 times. Respondents were most likely to report that they wished the providers had done testing (identified 192 times) or that providers had tried hard and did not give up (identified 170 times).

### Illustrative quotes about the diagnostic aftermath shared with patient co-authors

In open-ended comments, survey respondents described post-event response experiences, experiences of diagnostic problems that were not acknowledged, validated, or followed up on in their own care or for future patients. As an example, we shared the following verbatim quote with patient co-authors for discussion:

Well, initially, on the initial phone call, after I called, and then later, when it went through other channels there, less so. They tried to invalidate what I said and invalidate what their own people said on the phone with me, and that's what led to the back and forth emails, until I finally got them to relent and – I mean, all I got out of it was from a financial standpoint, but whether they did anything on that end to correct anything, highly doubtful.

In exploring post-event resolution experiences, survey respondents explained how they perceived the liability culture of medicine and clinician orientation to protect one another undermined efforts to respond to mishaps. For discussion of this preliminary finding, we shared the following passage for co-authors' consideration:

When no one answered my letter about what happened, it made me reluctant to use this practice. Since they are the only one of this type in this area (urogynecology) and I have ongoing issues, I did eventually go back to them. However, I missed annual checkups because I was upset at the way I was treated at the time [and] so what they did, they had one of the administrators of the hospital, she said she was going to file a complaint and I never heard anything. I never heard the results of the complaint. I think it was just a smoke screen, you know, just trying to I guess placate me and not really do anything. So they didn't really do anything. And you know, doctors, they will look out for each other, because they know that something like that could happen to them. It's easy to make a mistake and your career could be ruined. So I think they wanted to protect themselves and that's pretty much what happened.

### Patient co-author insight on mixed-methods results

Table 2 describes the insights patient co-authors offered in responding to the quantitative and qualitative findings, including additional insights not described in the text. The finding of the mishap not being acknowledged by the health system was unsurprising to the patient co-authors, and resonated with their own experiences and those they knew about from their patient and family networks. They noted that there is a perceived pressure for clinicians not to admit responsibility for a mishap, and that this perceived pressure was highly correlated with the significance of the mishap (i.e., more serious mishap, less likelihood to admit responsibility). It was also noted that while there are mandates in place that require acknowledgement of medical errors, application of these mandates in practice may be inconsistent given the complexity of diagnostic errors. That the majority of the respondents who wanted an apology did not receive one was also unsurprising to patient co-authors, who also emphasized the value of a sincere apology.

**Table 2:** Quantitative and qualitative emerging findings on post-event response and resolution experiences, and patient Co-authors insights.

Issue	Findings	Patient insights
Acknowledgement of Mishap	- Over a third of respondents reported that someone in the healthcare setting where the mistake occurred acknowledged the mistake (35.9%, 432/1,204). - “Did nothing” is the most commonly identified qualitative response (identified 325 times) to the question on what clinicians did after recognition of the problem or mistake.	- Difficulties in admitting fault and learning from mistakes, particularly in the context of doctors and hospitals harms patients and their care partners. - The pressure to never admit fault was identified as a significant issue. - Noted that such failures to respond were typical. - Noted a need for establishing protocols for acknowledging diagnostic errors and preventing future occurrences
Apology	- Excluding those who reported that an apology was not necessary (*n* = 377), only one-third of respondents reported receiving an apology (33.0%, 275/839).	- People want a sincere apology and assurance that measures are being taken to prevent similar incidents from happening again.
Perception that actions were seldom taken to prevent future issues	- Of the patients who did not report the mishap, almost half did not report because they did not think it would do any good (43.8%, 232/530). - Qualitative responses described a sense that no actions were taken to prevent the same mistakes or problems from happening to other patients	- Emphasized the importance of having a system in place to support such an approach, including access to necessary resources and regular follow-ups. - Clinical teams’ busy schedules and an increased number of emails from a variety of sources often lead to default responses, possibly contributing to a resistance to change to a system where time is taken to recognize, acknowledge, and act on patient and care partner reported issues. - Noted that providers sometimes seemed to feel stressed at the idea that patients should feel empowered to ask questions and understand their rights and options.
Fear of legal liability is preventing accountability	- About one-fifth did not report because they did not want to get anyone in trouble (17.4%, 92/530). - Qualitative responses described a perception that clinicians would “cover” for each other and did not want to validate respondents’ recognition of the problem or mistake.	- Noted fear of liability as a barrier to open communication. - It is understood concerns about liability exist, but they should not impact their patient care. - Emphasized that financial issues should be a last consideration, as they arise from failures in patient care and follow-up. - Fear of adverse consequences for themselves discourages doctors from providing feedback (e.g., there is a sense that clinicians do not want to acknowledge another clinician's error and report the error to them, as this may lead to negative repercussions for them). - Suggested incorporating training into medical education about mistake disclosure and accountability, and to shift away from the culture of covering up.

The patient co-authors were interested in the quantitative finding of patients not reporting mishaps because patients did not think it would do any good, and they resonated with the qualitative findings of patients reporting a mishap and perceiving that the health system did nothing to address the root causes of their mishap to prevent future harm to other patients. The importance of actions being taken to prevent future mishaps was viewed as particularly significant. Patient co-authors discussed how acting on patient-reported mishaps appropriately, so root causes could be identified and addressed, requires a health system to devote dedicated resources. They emphasized that having a person to respond to patient feedback and take phone calls meant little if that person did not also have resources to act upon what they heard. Patient co-authors viewed this exchange as a shared endeavor – when a patient reports a mishap, that patient should also be capacitated to request what changes they believe would be helpful to prevent future mishaps.

The fear of malpractice lawsuits was discussed as a barrier to openly communicating with patients when a mishap occurs. One patient co-author emphasized they consider a malpractice lawsuit to mean that the health system failed at taking steps to openly communicate and act on a patient-reported mishap; the malpractice lawsuit is a patient's last resort when they feel the mishap will not otherwise be recognized, including with actions taken to prevent mishaps happening to other patients. The other patient co-author pointed out that malpractice lawsuits are heavily emphasized in medical education training and practice, yet malpractice lawsuits themselves are relatively rare, and that training on mishap disclosure and accountability would not only do more to gain patients' trust and improve outcomes and patient safety, but also potentially prevent both mishaps and lawsuits from occurring. Both patient co-authors emphasized the need for accountability over issue avoidance when fears of a malpractice lawsuit override reality. Patient co-authors felt financial consequences to health systems arise from inaction, rather than from addressing patient concerns: the health systems having to manage financial issues resulting from a problem or mistake were seen as something that would only occur if health systems had not taken the appropriate actions to acknowledge and act on the identified problem.

### Future-facing recommendations on micro-, meso-, and macro-level

Together as an authorship team, we discussed future-facing recommendations at the micro-, meso-, and macro-levels. On a patient-level (referred to as the micro level for individual actions), there is a need to raise patient awareness of their rights to report mishaps in care. To our knowledge, most United States' health systems have an office specific to patient experience, where problems can be reported and, when possible, addressed, and the existence of this office and its role should be known by every patient. When patients report a problem, they should be prepared to articulate their desired resolution from the health system, including actions to be taken to prevent future problems. Individual clinicians and clinical teams can also consider the value of openly communicating with patients, and acknowledging when they perceive that there may have been a mishap in their care.

On a meso level, health systems should take steps to build cultures of accountability in their processes for error remediation. Adequate resources are essential for gathering patient feedback on diagnostic process breakdowns and implementing solutions at all levels within the healthcare delivery system. In particular, guidelines are needed on how to respond when a mistake or breakdown happens that is not necessarily attributable to any one person or action, but nonetheless impacted patient care; not acknowledging these issues to the patient leads to diminished trust.

On a macro level (meaning external influences on meso and micro levels), the patient co-authors noted that the Centers for Medicare and Medicaid Services (CMS) require healthcare systems to have mechanisms for patients to report mistakes. However, the fact that issues around diagnosis, often occur over time and across settings without a clear “problem owner” was identified as a potential reason why healthcare systems may be less likely to receive and manage reports of diagnostic mistakes. They noted that healthcare systems could benefit from better guidance on how best to enable reporting, and what actions to take on a problem or mistake, including the important step of closing the loop with the patient.

## Discussion

We present our learnings from working with patient co-authors to interpret quantitative and qualitative findings from a nationally representative survey on diagnostic problems and mistakes. These results suggest a path for “what to do next” on the patient, health system, and policy levels (also known as the micro, meso and macro levels) in response to insights from the experiences of many survey respondents. By employing the quantitative weight of these national estimates with accompanying qualitative illustrative quotes as motivators for changes to improve diagnosis in alignment with principles of high reliability organizing, the paper concludes with potential actions at multiple levels which were informed by patient advocate co-authors.

Patient co-authors identification of priority area for further analysis, and then leading our interpretation of the data, widened our understanding of the findings and contributed to a more comprehensive set of potential early solutions. Not only did the patient co-authors have their own firsthand experiences with diagnostic mishaps, they also regularly counsel others through their experiences with medical errors, and thus had wide knowledge of typical patient experiences in the aftermath of problems and mistakes. The patient co-authors' interpretation of analyses described in this paper will lay the foundation for future research by our study team focused on better understanding current practices in the aftermath, and how to improve.

High reliability organizing emphasizes deference to those closest to the process (10). There is no one closer to the diagnostic process than the patient (18). Yet, patients remain largely excluded from health care governance, including how mishaps are identified and managed (19). This exclusion not only further allows unacceptable practices, including not acknowledging a mishap once it has occurred, but also causes health systems to lose out on the substantial learnings enabled by patient inclusion. For example, it was continually emphasized that the very basics of patient communication - sincerely listening to the patient and acknowledging what they perceived as a mishap in their care, seem to have been lost by many healthcare sites. Future research that examines best practices in acknowledging a diagnostic mishap to the patient may benefit diagnostic quality overall.

### Limitations

This work is not without limitations. Two patient co-author insights were chiefly gathered over 2 one-hour meetings (one hour-long meeting with both patient co-authors, and a subsequent one-hour meeting with each patient co-author separately). The findings of our study are complex and further time could have been spent with a larger number of patient advocates to gather a broader range of perspectives. Both the quantitative and qualitative analysis conducted of the patient-reported aftermath were preliminary, and we thus may be missing other important findings.

## Conclusion

In conclusion, the urgent need for improved diagnosis in healthcare underscores the critical role of patient engagement. Patients possess invaluable insights into diagnostic breakdowns and adverse experiences that are often overlooked in traditional quality improvement efforts. By embracing patient engagement as a cornerstone of high reliability organizing, healthcare systems can harness the expertise of those closest to the work, fostering a culture of safety and driving meaningful improvements in diagnostic accuracy and patient outcomes.

## Data Availability

The datasets presented in this article are available upon reasonable request to the corresponding author. Requests to access the datasets should be directed to Kelly Gleason, kgleaso2@jhu.edu.
