# Supplementary material for: Patient-informed exploration of the aftermath of a diagnostic problem or mistake based on results of a national survey
Source: Front Health Serv. 2024 Nov 28;4:1474073. doi: 10.3389/frhs.2024.1474073 (PMC11634834; doi:10.3389/frhs.2024.1474073)
Supplement: Supplementary file 1 [file Datasheet1.pdf]

Supplementary Material

Screening question

In the past four years – that would be since about the start of 2019 – was there a mistake or problem with the diagnosis of a medical condition?

RESPONSE OPTIONS:

01 In your own care

02 In the care of someone else living in your household

03 In the care of someone in your family living outside of the household

04 Someone else not in your family or not living in your household

Q1 Did more than one diagnostic mistake or problem happen to [you/them] in the past four years?

RESPONSE OPTIONS:

01 Yes

02 No

[SHOW IF Q1=1]

Though you/they have experienced more than one mistake or problem with a diagnosis in the last four years, please think of <u>the one you remember best</u> when answering the next set of questions.

Q2AA Approximately what month and year [did this/did this most memorable] diagnostic mistake or problem first begin?

RESPONSE OPTIONS:

01 January

02 February

03 March

04 April

05 May

06 June

07 July

08 August

09 September

10 October

11 November

12 December

Q2AB Approximately what month and year [did this/did this most memorable] diagnostic mistake or problem first begin?

RESPONSE OPTIONS:

01 2023

02 2022

03 2021

04 2020

05 2019

06 2018

07 2017 or earlier

Q2B Even though it began earlier, did the search for a correct diagnosis continue into 2019?

RESPONSE OPTIONS:

01 Yes

02 No

Other survey questions included in this analysis:

Q3

Did the mistake or problem with diagnosis happen to:

RESPONSE OPTIONS:

01 You

02 Your spouse

03 A family member who lives in your home. *Please specify your relationship with this person:* ______

04 A family member who lives outside of your home. *Please specify your relationship with this person:*_______

05 A person living in your home who is not related to you

06 A person living outside your home who is not related to you

Q3A

Were you responsible for making decisions about this person's care at the time the diagnostic mistake or problem occurred?

RESPONSE OPTIONS:

01 Yes

02 No

Q3B

As the diagnostic mistake or problem was emerging, were you closely accompanying the patient on visits to the doctor or regularly discussing those visits with them after the fact?

RESPONSE OPTIONS:

01 Yes

02 No
